# DualStream-RTNet: A Multimodal Deep Learning Framework for Grape Cultivar Classification and Soluble Solid Content Prediction

**DOI:** 10.3390/foods15061095

**Published:** 2026-03-20

**Authors:** Zhiguo Liu, Yufei Song, Aoran Liu, Xi Meng, Chang Liu, Shanshan Li, Xiangqing Wang, Guifa Teng

**Affiliations:** 1 College of Information Science and Technology, Hebei Agricultural University, Baoding 071001, China; sjzlzg@163.com; 2 Hebei Key Laboratory of IoT Blockchain Integration, School of Future Information Technology, Shijiazhuang University, Shijiazhuang 050037, China; 13223450412@163.com (A.L.); mengxi19930511@163.com (X.M.); lliuchang1122@163.com (C.L.); liss@sjzc.edu.cn (S.L.); wangxiangqing@sjzc.edu.cn (X.W.); 3 School of College of Computer and Cyber Security, Hebei Normal University, Hebei Provincial Key Laboratory of Network & Information Security, Hebei Provincial Engineering Research Center for Supply Chain Big Data Analytics & Data Security, Shijiazhuang 050025, China

**Keywords:** multimodal fusion, hyperspectral imaging, multi-task deep learning, RGB-HSV feature fusion

## Abstract

Accurate and non-destructive evaluation of grape quality is crucial for intelligent viticulture, yet most existing approaches address cultivar classification and soluble solid content (SSC) prediction as independent tasks based on single-modality data, limiting robustness and practical applicability. This study proposes DualStream-RTNet, a unified multimodal deep learning framework that simultaneously performs grape cultivar classification and SSC prediction by integrating RGB-HSV fused images and PCA-compressed hyperspectral spectra. The dual-stream architecture enables the complementary learning of external chromatic–textural cues and internal physicochemical information, while a Transformer-enhanced fusion module strengthens global representation and cross-modal correlation. A dataset of 864 berries from five grape cultivars was used to validate the model. DualStream-RTNet achieved 93.64% classification accuracy, outperforming ResNet18 and other CNN baselines, and produced more compact and consistent confusion-matrix patterns. For SSC prediction, it consistently yielded the highest performance across cultivars, with R2p values up to 0.9693 and RMSE as low as 0.2567, surpassing the PLSR, SVR, LSTM, and Transformer regression models. These results demonstrate the superiority of the proposed framework in capturing both visual and spectral characteristics. DualStream-RTNet provides an efficient and scalable solution for comprehensive grape quality assessment, offering strong potential for real-time sorting, precision grading, and smart agricultural applications.

## 1. Introduction

Grapes are among the most economically significant fruit crops worldwide and play a crucial role in both fresh consumption and processing industries due to their desirable sensory attributes, nutritional value, and market versatility [1,2,3]. Accurate grape cultivar identification and precise assessment of soluble solid content (SSC), a key indicator of sweetness and overall flavor quality, are essential for quality grading, varietal authentication, harvest timing optimization, and postharvest supply-chain management [4,5,6]. Reliable cultivar recognition ensures varietal purity and market transparency, while SSC measurement directly reflects consumer acceptance and commercial value. Consequently, efficient and accurate grape quality evaluation is a fundamental requirement for intelligent viticulture and precision agriculture systems.

Conventional approaches for grape quality assessment primarily rely on manual visual inspection and destructive biochemical analysis. Visual inspection is subjective and highly dependent on expert experience, whereas SSC measurement typically requires juice extraction and laboratory-based refractometry, rendering it time-consuming, destructive, and unsuitable for large-scale or real-time applications [7,8,9]. These limitations have motivated extensive research into non-destructive sensing techniques that can provide rapid, objective, and repeatable quality evaluation. Among these techniques, computer vision and hyperspectral sensing have emerged as two of the most widely adopted modalities for external phenotype recognition and internal quality analysis, respectively [10,11,12].

In recent years, deep learning-based computer vision methods have demonstrated strong capability in fruit appearance analysis, including grape cultivar classification using RGB images. Convolutional neural networks have been successfully employed to extract discriminative color, texture, and morphological features, enabling automatic recognition of grape varieties under controlled imaging conditions [13,14,15]. However, RGB-based methods primarily capture surface-level visual information and are inherently limited in their ability to reflect internal physicochemical attributes. In parallel, hyperspectral imaging and Vis–NIR spectroscopy have been extensively investigated for non-destructive SSC prediction, leveraging spectral absorption characteristics related to sugar composition and water content [16,17,18,19]. Regression models based on PLSR, SVR, and, more recently, deep learning architectures such as LSTM and Transformer models have achieved encouraging performance in modeling complex spectral–chemical relationships.

Despite these advances, most existing studies treat grape cultivar classification and SSC prediction as independent tasks and rely on single-modality inputs [9,13]. Image-based approaches focus exclusively on external appearance, while spectral-based methods are designed solely for internal quality estimation. Such unimodal and single-task paradigms fail to exploit the intrinsic complementarity between visual phenotypes and physicochemical properties. Moreover, deploying separate models for classification and SSC prediction introduces computational redundancy, increases system complexity, and limits practical deployment efficiency in intelligent vineyard monitoring and automated sorting systems [20,21,22]. Although recent studies suggest that multimodal integration can enhance non-destructive fruit quality assessment by combining heterogeneous information sources [23], truly unified, end-to-end frameworks capable of simultaneously performing grape cultivar recognition and SSC estimation remain insufficiently explored.

Multimodal learning has therefore emerged as a promising paradigm for agricultural sensing applications [24]. By integrating visual, spectral, and sometimes environmental data, multimodal approaches aim to capture complementary information that cannot be obtained from a single modality alone. Existing multimodal fusion strategies can generally be categorized into three main types: early fusion [25], late fusion [26], and attention-based fusion [27]. Early fusion combines raw or low-level features from multiple modalities before feature extraction, enabling joint representation learning but often suffering from noise sensitivity and modality imbalance. Late fusion integrates modality-specific predictions at the decision level, offering flexibility but limiting cross-modal interaction during feature learning. More recently, attention-based fusion methods—particularly those employing Transformer architectures—have demonstrated superior capability in modeling cross-modal correlations through adaptive weighting and global dependency learning [28,29].

In the context of fruit quality assessment, several studies have applied multimodal fusion to improve classification or regression performance. However, most reported fusion frameworks are still designed for single-task objectives and typically adopt relatively shallow fusion mechanisms [9,13,18,19,22,30]. The potential of combining multimodal learning with multi-task modeling has not been fully exploited. Multi-task learning enables multiple related tasks to be jointly optimized within a shared framework, facilitating shared representation learning, reducing model redundancy, and improving generalization through cross-task knowledge transfer. In agricultural and food quality applications, Multi-task learning has been explored for joint prediction of maturity indices, defect detection, and physicochemical parameters [31,32]. Nevertheless, these attempts remain limited in scope, and unified multimodal multi-task architectures that simultaneously integrate heterogeneous sensing information and address both categorical and continuous prediction tasks are still scarce.

To address these challenges, this study proposes DualStream-RTNet, a unified multimodal and multi-task deep learning framework for simultaneous grape cultivar classification and SSC prediction. The proposed architecture adopts a task-oriented dual-stream design, in which RGB-HSV fused images and PCA-compressed hyperspectral spectra are processed through parallel CNN–Transformer branches tailored to their respective tasks. For each task, local feature extraction and global contextual modeling are jointly performed, enabling comprehensive representation learning. A learnable softmax-weighted fusion mechanism is further introduced to adaptively balance the contributions of different feature streams, enhancing cross-modal interaction while maintaining flexibility. By integrating multimodal fusion and multi-task learning within a single end-to-end architecture, DualStream-RTNet effectively reduces model redundancy and improves both classification and regression performance. This unified framework provides a more practical and scalable solution for intelligent viticulture, supporting simultaneous visual inspection and internal quality assessment for real-world applications.

## 2. Methods

### 2.1. Acquisition of Sample Images and Spectral Data

**Figure 1:**
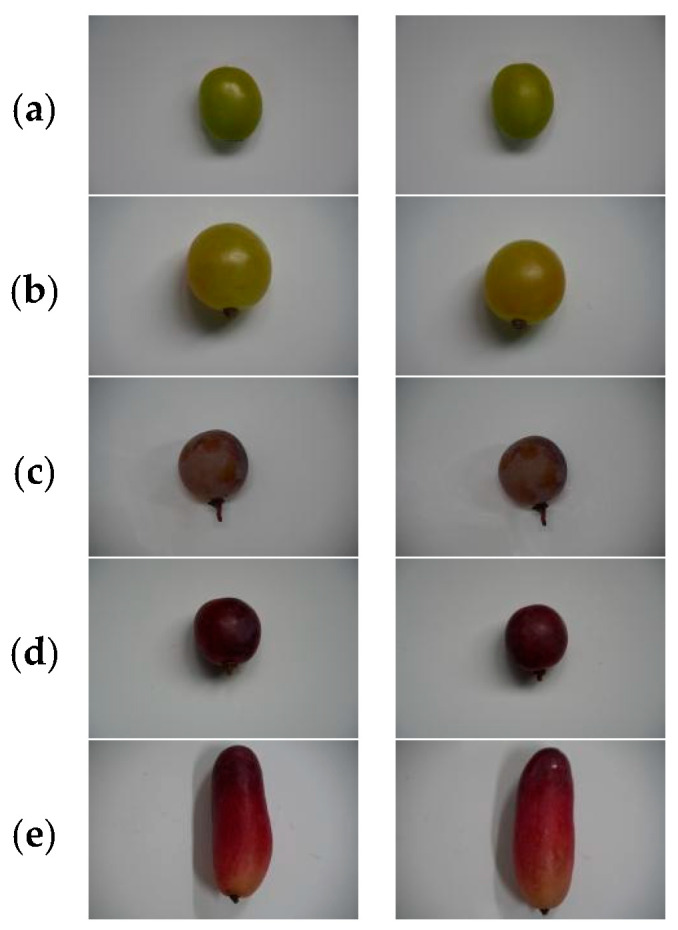
Example Samples of the Five Grape Cultivars ((**a**): Shine Muscat, (**b**): Yubo No. 2, (**c**): Longyan, (**d**): Red Globe, (**e**): Meiren Zhi).

The experimental samples used in this study were collected in Huailai County, Zhangjiakou City, Hebei Province, China. Located in the northwestern part of the Beijing–Tianjin–Hebei region, Huailai is recognized as one of the most suitable areas for high-quality grape cultivation in northern China. The region features a temperate continental monsoon climate characterized by ample sunlight, large diurnal temperature variations, low rainfall during the fruit maturation period, and well-drained sandy loam soils. These conditions promote balanced sugar accumulation, enhanced aroma development, and stable fruit coloration, making Huailai an ideal location for producing diverse table grape cultivars with distinctive physicochemical properties. Sample collection was conducted from 1 October to 25 October 2025, coinciding with the commercial harvest period of all target cultivars. In accordance with the Chinese national standard GB/T 30763-2014 “Guideline for grade of agricultural products” [33], berries were selected based on uniform maturity, absence of mechanical damage, freedom from disease or insect infestation, and representative coloration for each cultivar. A total of 864 grape berries were collected across five varieties (Shine Muscat, Yubo No. 2, Longyan, Red Globe, and Meiren Zhi), The grape samples of the five varieties were shown in Figure 1. All samples were immediately transported to the laboratory under temperature-controlled conditions to minimize physiological changes prior to imaging and spectral acquisition.

After transportation to the laboratory, all grape samples underwent a standardized imaging and spectral acquisition procedure to ensure data consistency and reproducibility. Image acquisition was performed under uniform indoor environmental. A high-resolution Sony ILCE-6400L mirrorless camera equipped with a Sony E PZ 18–105 mm F4 G OSS lens was employed for static image capture (Sony, Tokyo, Japan). The imaging background consisted of a matte white PVC board with high diffuse reflectivity, which enhanced foreground–background separation and facilitated subsequent image preprocessing. During image capture, each berry was positioned on the fixed PVC surface and photographed from a vertical top-down perspective at a constant distance of 40 cm. The camera was operated in fully manual mode with fixed parameters (shutter speed: 1/250 s; aperture: F/5.6; ISO: 100; focal length: 50 mm) to ensure uniform exposure across the entire dataset. White balance settings were also held constant to avoid chromatic bias. Each berry was imaged from two orientations (front and back), resulting in a total of 1728 high-resolution RGB images, all saved in lossless PNG format. Prior to model training, all images were uniformly cropped and resized to 224 × 224 pixels, followed by pixel-level intensity normalization to meet the input requirements of deep learning models.

**Figure 2:**
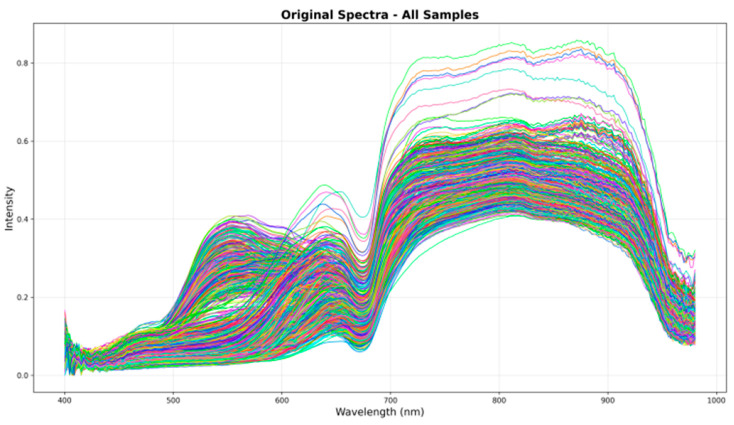
Spectral Images of all Grape Samples.

In addition to imaging data, hyperspectral measurements were acquired to support soluble solid content (SSC) prediction. A FIGSPEC FS-13 hyperspectral imaging system (Caimatech Co., Ltd., Shenzhen, China) was used for spectral acquisition, covering a wavelength range of 390–1000 nm with a spectral resolution of 2.5 nm, resulting in approximately 300 bands. The hyperspectral camera was positioned vertically at a fixed height of 20 cm above the sample stage inside an enclosed imaging chamber. Illumination was provided exclusively by eight halogen lamps arranged at 45° angles to the sample, ensuring stable and uniform lighting conditions. Before each acquisition session, the system was preheated for 5 min and calibrated using a standard white reference panel to ensure spectral accuracy. For each grape berry, the hyperspectral system captured the reflectance information from the entire visible surface within the camera’s field of view, rather than from discrete sampling points. The acquired spatial–spectral data cube was then averaged across the valid fruit-region pixels to generate a single representative spectral curve for each sample. This full-surface averaging approach effectively reduced random noise introduced by local texture variations and ensured that the resulting spectrum accurately reflected the overall optical properties of the berry. Bands with excessive noise near both ends of the spectrum (390–400 nm and 980–1000 nm) were removed, resulting in a final effective wavelength range of 400.46–979.62 nm used for subsequent analysis [30]. The spectral curves of all samples are presented in Figure 2.

To ensure a balanced and fair evaluation of model performance, the 864 grape samples were partitioned according to the five target cultivars included in this study. The dataset was stratified by grape variety to preserve the original class distribution during model training and testing. As shown in Table 1, the final number of samples for each cultivar was as follows: 164 for Shine Muscat (C1), 200 for Yubo No. 2 (C2), 204 for Longyan (C3), 212 for Red Globe (C4), and 84 for Meiren Zhi (C5). Each class was then divided into training and test sets following a consistent partitioning ratio, resulting in 691 samples for training and 173 for testing. Stratified sampling [34] was adopted to ensure that the proportional representation of each grape variety remained unchanged across the subsets, thereby supporting reliable and unbiased model evaluation.

### 2.2. Extraction of RGB and HSV Statistical Features

In addition to raw image data, six fundamental RGB statistical features were extracted from each grape sample to characterize its overall chromatic and surface reflectance properties. Specifically, the mean intensity values of the red, green, and blue channels (R_MEAN, G_MEAN, B_MEAN) were calculated to quantify the global color tendencies of the berry surface, while the corresponding standard deviations (R_STD, G_STD, B_STD) were computed to reflect color variability and surface uniformity. These metrics provide a compact and informative representation of the fruit’s appearance, capturing both dominant chromatic information and fine-scale texture variations. As shown in Table 2, all five grape cultivars exhibited distinct RGB distribution patterns, with color means and variances differing across varieties such as Shine Muscat, Yubo No. 2, and Red Globe. These statistical descriptors were subsequently used as auxiliary features to support model development and further analyze inter-varietal visual differences.

To further highlight the inter-cultivar chromatic differences, the class-wise mean values of the extracted RGB statistical features were calculated, as summarized in Table 3. The results show noticeable variations in both channel intensity and dispersion characteristics among the five grape cultivars. For example, cultivars such as Shine Muscat and Red Globe exhibit relatively higher R_MEAN and G_MEAN values, whereas certain cultivars display larger standard deviations, indicating increased surface color variability. These averaged statistical descriptors provide a clearer quantitative comparison of visual appearance across cultivars and further confirm the existence of distinguishable inter-varietal color distribution patterns, which can effectively support downstream classification and multimodal feature analysis.

Following the computation of RGB-based descriptors, additional statistical features were extracted from the HSV color space to capture perceptually meaningful chromatic variations in grape surfaces. By separating hue (H), saturation (S), and value (V) components, the HSV representation provides a more intuitive and perceptually interpretable description of fruit surface color, while offering improved robustness to illumination variations compared with RGB-based descriptors. Consequently, HSV features are particularly suitable for quantifying cultivar-dependent chromatic differences in grape images. For each grape image, the mean values of the H, S, and V channels (H_MEAN, S_MEAN, V_MEAN) were calculated to represent the dominant hue tendency, chromatic intensity, and brightness level, respectively. Correspondingly, the channel-wise standard deviations (H_STD, S_STD, V_STD) were computed to assess the variability and uniformity of these attributes across the berry surface. As shown in Table 4, the five grape cultivars exhibited distinct HSV statistical patterns, with varieties such as Red Globe tending to show higher hue dispersion, while Shine Muscat typically displayed lower saturation variance. These HSV-based descriptors complement the RGB statistics by providing a more comprehensive characterization of grape color dynamics, thereby enriching the feature space for subsequent model training and comparative analysis.

To provide a clearer quantitative comparison of color characteristics across cultivars, the class-wise mean values of the HSV statistical descriptors were further calculated, as summarized in Table 5. The results indicate noticeable inter-cultivar differences in both chromatic intensity and variability. For instance, Yubo No. 2 and Meiren Zhi exhibit relatively higher H_MEAN values, suggesting a tendency toward different dominant hue distributions compared with the other cultivars, while variations in S_STD and V_STD reflect differences in color saturation consistency and brightness uniformity across berry surfaces. These averaged HSV statistics complement the RGB-based analysis and further confirm the presence of distinguishable color distribution patterns among the five grape cultivars, providing informative auxiliary cues for subsequent multimodal feature integration and model training.

### 2.3. Acquisition of Soluble Solid Content (SSC) Measurements

In addition to color features, the soluble solid content (SSC) of each grape berry was measured to provide accurate reference values for regression modeling. SSC determination was conducted using a PAL-1 digital refractometer (ATAGO, Tokyo, Japan), which offers a Brix measurement range of 0.0–53.0% with an accuracy of ±0.2% and includes an automatic temperature compensation system to eliminate temperature-induced measurement errors. Prior to use, the device was calibrated to zero. Grape juice was extracted using a portable stainless-steel manual juicer, filtered, and approximately 3 mL of juice was transferred onto the refractometer prism using a graduated pipette. After allowing the instrument to stabilize for three seconds, the SSC reading was recorded. Each sample was measured three times, and the arithmetic mean was taken as the final SSC value to ensure measurement stability and reduce random error [35,36]. The resulting SSC distributions for all grape cultivars are summarized in Table 6, revealing clear inter-varietal differences, with cultivars such as Shine Muscat and Red Globe exhibiting generally higher sweetness levels, while Meiren Zhi showed broader variability. These high-precision SSC measurements served as the ground-truth labels for the regression component of this study.

**Figure 3:**
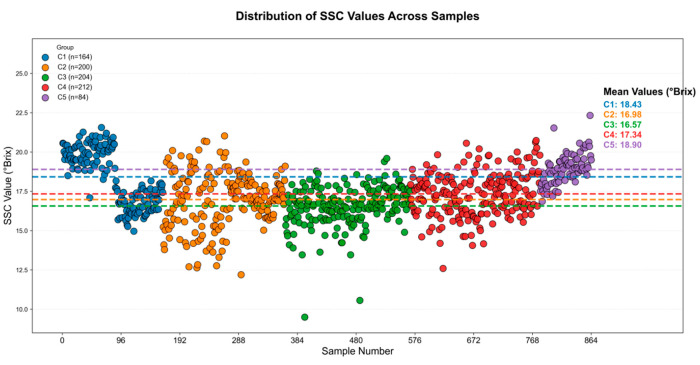
Distribution of SSC Across Five Grape Cultivars.

The overall distribution of SSC values for the five grape cultivars is presented in Figure 3, where each point denotes the mean SSC of an individual berry and dashed horizontal lines indicate cultivar means. Quantitatively, the group means are C1 = 18.43 °Brix, C2 = 16.98 °Brix, C3 = 16.57 °Brix, C4 = 17.34 °Brix, and C5 = 18.90 °Brix. Several clear patterns emerge. Shine Muscat (C1) forms a dense cluster at the upper end of the SSC scale with relatively low dispersion, indicating stable and consistently high sugar accumulation. Yubo No. 2 (C2) and Longyan (C3) show broader, more diffuse distributions centered at lower mean values, reflecting greater within-cultivar variability and the presence of low-SSC outliers (particularly in C3). Red Globe (C4) occupies an intermediate range with moderate clustering around its mean, suggesting fairly consistent but not maximal sweetness. Meiren Zhi (C5) has the highest mean and also exhibits a wider spread and several high-SSC samples, indicating both elevated average sweetness and larger internal variation. Such well-separated SSC patterns not only validate the necessity of cultivar-specific modeling, but also provide a meaningful foundation for evaluating the SSC prediction performance of the proposed DualStream-RTNet framework.

### 2.4. DualStream-RTNet Framework for Grape Cultivar Classification and SSC Prediction

**Figure 4:**
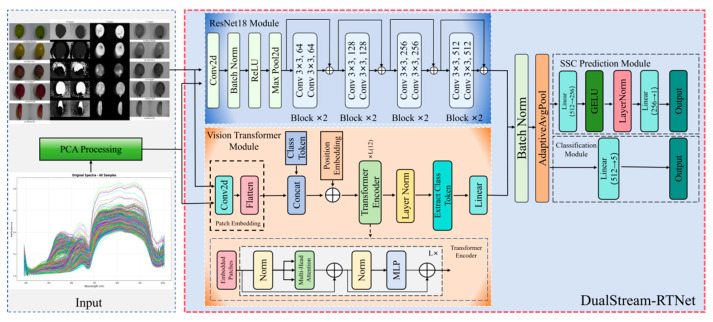
DualStream-RTNet Network Structure for Grape Classification and SSC Prediction.

Most existing grape quality assessment methods are designed to address either cultivar classification or soluble solid content (SSC) prediction independently and typically rely on single-modality inputs. Such approaches fail to fully exploit the complementary information between external visual cues and internal physicochemical properties, thereby limiting robustness and generalization. To address these limitations, this study proposes DualStream-RTNet, a unified multimodal and multi-task deep learning framework that simultaneously performs grape cultivar classification and SSC prediction within a single end-to-end architecture. The network architecture of DualStream-RTNet is shown in Figure 4.

The core innovation of DualStream-RTNet lies in its task-specific dual-stream representation learning design, in which different data modalities are processed independently according to their target tasks, while each task itself is modeled using a dual-stream architecture. Specifically, for grape cultivar classification, fused RGB–HSV images are fed into a dual-stream network composed of a CNN-based ResNet18 branch and a Transformer-based branch, enabling simultaneous extraction of local color–texture features and global contextual representations. For SSC prediction, PCA-reduced hyperspectral data are likewise processed through a parallel dual-stream structure, allowing the model to capture both localized spectral patterns and long-range wavelength dependencies. By maintaining a consistent dual-stream paradigm across tasks while separating modality inputs according to their functional relevance, DualStream-RTNet effectively avoids cross-task interference and preserves modality-specific discriminative power. This design enables comprehensive feature modeling within each task and ensures that both grape cultivar classification and SSC prediction benefit from complementary local–global representations, offering a flexible yet unified solution that surpasses conventional single-stream or single-task architectures.

To further enhance multimodal synergy, a learnable softmax-weighted fusion mechanism is introduced to adaptively balance the contributions of the two feature streams. The fused representation is then shared by task-specific heads for cultivar classification and SSC regression, enabling effective knowledge sharing through multi-task learning. This design not only improves predictive accuracy and stability, but also reduces model redundancy, providing an efficient and robust solution for integrated grape quality evaluation.

### 2.5. Performance Evaluation Metrics of the Model

To comprehensively assess the performance of the proposed DualStream-RTNet in both grape cultivar classification and soluble solid content (SSC) prediction, two groups of quantitative evaluation metrics were employed. Classification performance was evaluated using Accuracy, Precision, Recall, and the Confusion Matrix, while SSC prediction was assessed using the coefficient of determination (R^2^) and the root mean square error (RMSE). These metrics collectively enabled a rigorous analysis of the model’s discriminative capability, generalization behavior, and predictive accuracy across tasks.

For the grape cultivar classification task, model performance was first evaluated using Accuracy, which reflects the proportion of correctly classified samples among all samples. Accuracy is computed as

(1) $$Accuracy=\frac{TP+TN}{TP+TN+FP+FN}$$

where *TP*, *TN*, *FP*, and *FN* denote true positives, true negatives, false positives, and false negatives, respectively. Although accuracy provides an intuitive global measure of performance, it may not fully capture class-wise behavior, particularly in the presence of class imbalance.

To overcome this limitation, Precision and Recall were adopted as complementary metrics. Precision measures the proportion of correctly predicted positive samples among all predicted positives, and is defined as

(2) $$\mathrm{Pr}ecision=\frac{TP}{TP+FP}$$

Recall (also referred to as sensitivity or the true positive rate) quantifies the model’s ability to correctly identify all actual positive samples:

(3) $$Recall=\frac{TP}{TP+FN}$$

Together, Precision and Recall provide a more detailed interpretation of class-specific performance and enable a nuanced evaluation of the model’s capability to distinguish among visually similar grape cultivars.

In addition, the Confusion Matrix was employed to visualize classification outcomes across all categories. This matrix highlights misclassification patterns and inter-class similarities, thereby offering valuable insights into model behavior and potential feature overlaps among cultivars.

For the SSC prediction task, model performance was evaluated using the coefficient of determination (*R^2^*) and the root mean square error (*RMSE*). The coefficient of determination, denoted as R^2^c for training and R^2^p for testing, is defined as

(4) $$R^{2}=1-\frac{\sum_{i=1}^{N}{\left(y_{i}-{\hat{y}}_{i}\right)}^{2}}{\sum_{i=1}^{N}{\left(y_{i}-\bar{y}\right)}^{2}}$$

where represents the total number of samples, is the ground-truth SSC value of the *i*-th sample, is the predicted value, and is the mean SSC across all samples. R^2^ reflects the proportion of variance in SSC explained by the model and thus indicates the goodness of fit.

RMSE, denoted as RMSE_C_ for training and RMSE_P_ for testing, measures the average deviation between predicted and true SSC values:

(5) $$RMSE=\sqrt{\frac{1}{N}\sum_{i=1}^{N}{\left(y_{i}-{\hat{y}}_{i}\right)}^{2}}$$

As a scale-dependent metric, RMSE provides an intuitive interpretation of prediction error magnitude. Together R^2^ and RMSE offer a robust and complementary evaluation of the model’s regression accuracy, enabling comprehensive assessment of the SSC prediction performance of DualStream-RTNet.

## 3. Results

### 3.1. Performance Comparison of Different Models Using RGB and HSV Inputs

To establish a comprehensive understanding of how different color representations influence grape cultivar classification, three commonly used convolutional neural network (CNN) architectures—ResNet18, MobileNetV3, and VGG16—were first evaluated using RGB images alone. The results shown in Table 7 provide an initial baseline for assessing model performance under single-color-space conditions.

As shown, ResNet18 outperformed the other architectures with a test accuracy of 80.35%, demonstrating balanced training–testing performance. In contrast, MobileNetV3 exhibited clear overfitting, while VGG16 showed limited discriminative capability. These findings confirm that RGB-only information is insufficient for capturing the subtle chromatic variations between grape cultivars.

To further examine the influence of alternative color representations, the same models were trained using HSV images. HSV separates chromaticity from luminance, providing additional sensitivity to color intensity and saturation. The results are summarized in Table 8.

Compared with RGB, HSV inputs led to improved test accuracy for ResNet18 (82.08%) and VGG16 (75.14%), indicating that HSV provides more discriminative color cues for cultivar separation. Nevertheless, single-color-space inputs still exhibited limited performance, suggesting that neither RGB nor HSV alone is sufficient for optimal recognition.

To further examine whether complementary chromatic cues could improve classification robustness, RGB and HSV images were fused to construct a six-channel RGB-HSV representation. The three CNN architectures were then retrained and evaluated under identical experimental conditions. The resulting performance metrics, summarized in Table 9, provide a rigorous assessment of each model’s capacity to leverage the fused color features for grape cultivar recognition. This comparative analysis offers a systematic evaluation of the generalization behavior of standard CNN architectures when supplied with enriched chromatic information.

Among all evaluated models, ResNet18 exhibited the most robust and well-balanced performance, achieving a training accuracy of 85.09% and a test accuracy of 84.39%. The close alignment between its precision (84.96%) and recall (84.39%) on the test set demonstrates the model’s ability to consistently distinguish among the five grape cultivars without bias toward any particular category. Importantly, the minimal performance gap between training and testing indicates an absence of overfitting, thereby establishing ResNet18 as a stable and reliable baseline for subsequent architectural enhancements developed in this study.

In contrast, MobileNetV3 demonstrated noticeable overfitting, characterized by a high training accuracy of 91.46% but a substantially lower test accuracy of 80.92%. Although MobileNetV3 integrates efficient depthwise separable convolution operations, its elevated training precision (89.47%) and significantly reduced test recall (80.92%) suggest limited capability in generalizing across samples with subtle color variations—a key challenge in grape cultivar classification. VGG16 yielded the lowest overall performance, reaching a test accuracy of 80.35%. Despite achieving moderate training metrics, its inferior generalization highlights the shortcomings of conventional deep CNN architectures lacking residual connections and modern design optimizations. The inability of VGG16 to effectively capture intertwined chromatic and textural cues further reinforces the need for more advanced feature extractors. Collectively, these results confirm that ResNet18 offers the optimal trade-off between complexity, representational strength, and generalization performance.

To further investigate the influence of network depth on the ability to extract discriminative RGB-HSV features, four ResNet variants—ResNet18, ResNet34, ResNet50, and ResNet101—were evaluated under the same experimental protocol. The training and testing results, summarized in Table 10, reveal notable performance differences associated with increased architectural depth.

**Figure 5:**
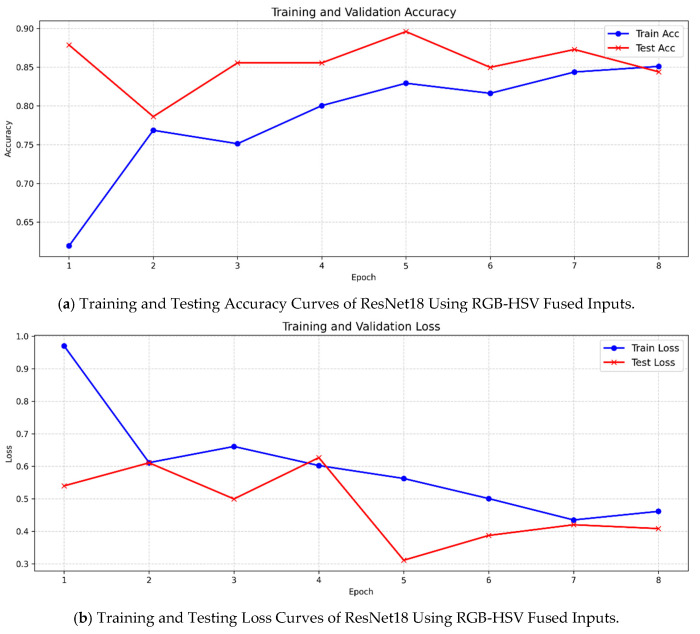
Training and Testing Accuracy and Loss Curves of ResNet18 Using RGB-HSV Fused Inputs.

**Figure 6:**
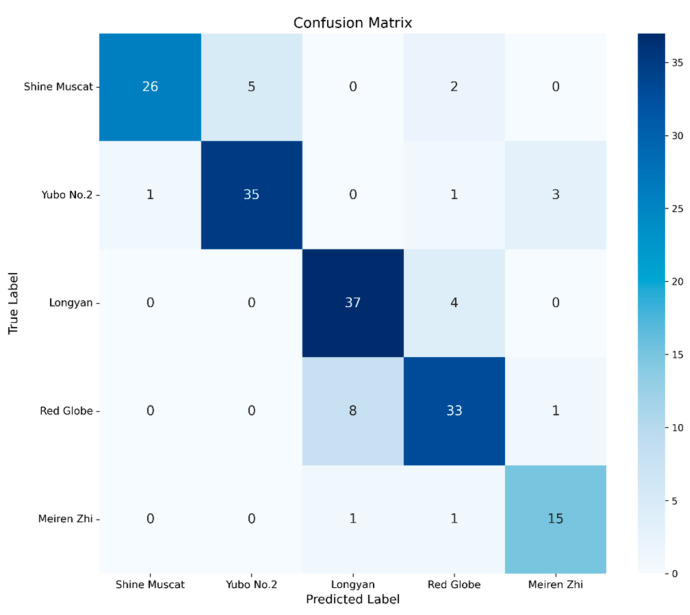
Confusion Matrix of ResNet18 for RGB-HSV-Based Grape Cultivar Classification.

ResNet18 again served as a strong baseline, exhibiting a balanced relationship between training accuracy (85.09%) and testing accuracy (84.39%). Its stable generalization confirms that shallow-to-moderate depth is sufficient for modeling the color-dominated characteristics of grape images. The training and testing accuracy and loss curves for ResNet18 are presented in Figure 5, while the corresponding confusion matrix is illustrated in Figure 6.

ResNet34 and ResNet50 demonstrated moderate improvements, with test accuracies of 83.24% and 85.55%, respectively. ResNet50 achieved the highest test accuracy among the four variants, suggesting that its deeper residual hierarchy yields slightly enhanced feature abstraction. However, the performance gains over ResNet18 remain marginal, indicating that further increasing depth offers diminishing returns for this task. In stark contrast, ResNet101 exhibited a pronounced overfitting trend. Although it reached a training accuracy of 85.67%, its test accuracy dropped sharply to 73.41%, with both precision and recall decreasing substantially. The significant performance gap highlights the limitations of excessively deep networks in scenarios where the discriminative cues are predominantly low-level chromatic patterns rather than high-level semantic structures. The overparameterization of ResNet101 likely results in suboptimal generalization given the dataset scale and task complexity.

### 3.2. Comparison of the Performance Between DualStream-RTNet and Standard ResNet18

**Figure 7:**
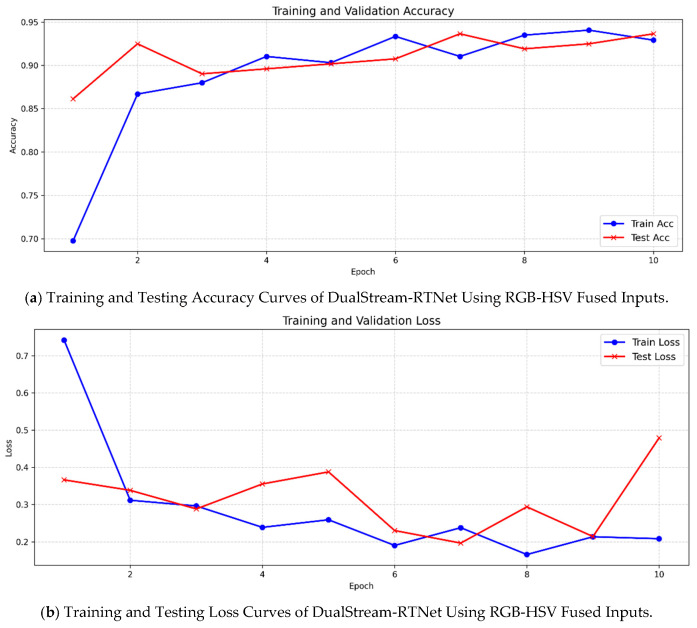
Training and Testing Accuracy and Loss Curves of DualStream-RTNet Using RGB-HSV Fused Inputs.

**Figure 8:**
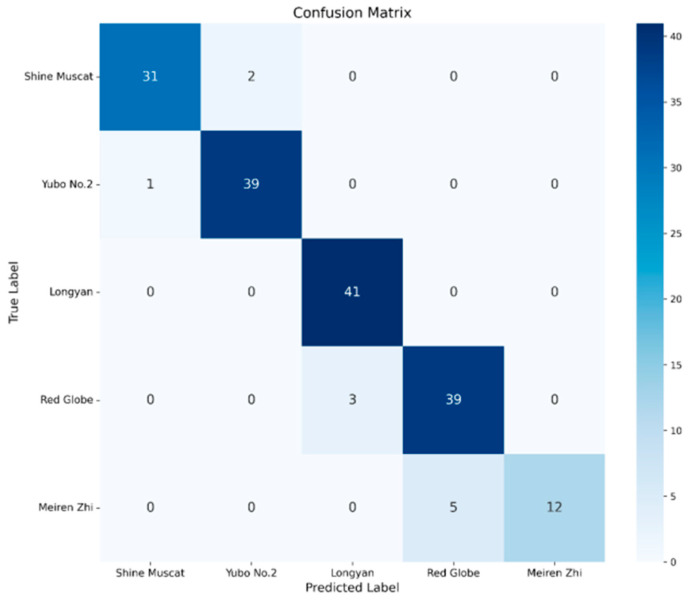
Confusion Matrix of DualStream-RTNet for RGB-HSV-Based Grape Cultivar Classification.

To comprehensively assess the effectiveness of the proposed DualStream-RTNet architecture, a direct comparison was conducted between DualStream-RTNet and the standard ResNet18 baseline under identical training configurations. As presented in Table 11, the proposed model achieves substantial improvements across all performance metrics, demonstrating its enhanced capacity for grape cultivar classification using RGB-HSV fused inputs. The training and testing accuracy and loss curves for DualStream-RTNet are illustrated in Figure 7, providing clear evidence of its superior convergence behavior. Additionally, Figure 8 visualizes the classification results of the DualStream-RTNet model through a confusion matrix, highlighting the model’s significant improvement in class-level discriminative capability.

ResNet18, serving as the baseline network, achieved a test accuracy of 84.39%, with corresponding precision and recall values of 84.96% and 84.39%, respectively. These results reflect the baseline model’s stable generalization performance but also reveal its limited ability to fully exploit the multimodal color information contained in the RGB-HSV representation. In contrast, DualStream-RTNet achieved significantly superior results, attaining a test accuracy of 93.64%, along with precision and recall values of 93.90% and 93.64%, respectively. These improvements represent an increase of more than 9 percentage points over the baseline across all three evaluation metrics. Such substantial gains highlight the clear advantages brought by the dual-stream feature extraction and Transformer-based fusion mechanisms incorporated into the proposed architecture.

The performance enhancements can be attributed to two key design elements. First, the dual-stream structure enables the network to independently extract and integrate complementary features from RGB and HSV color spaces, thereby improving its sensitivity to subtle chromatic and textural variations among grape cultivars. Second, the integrated Transformer module effectively captures long-range dependencies and global contextual relationships that conventional CNN architectures struggle to model, leading to more robust and discriminative feature representations.

Moreover, DualStream-RTNet also demonstrated a higher training accuracy (92.91%) compared to ResNet18 (85.09%), indicating more efficient convergence and stronger feature-learning capacity during training. Collectively, these findings provide compelling evidence that DualStream-RTNet offers a substantially improved classification framework and serves as a more powerful alternative to the standard ResNet18 architecture for multimodal color-based grape cultivar identification.

### 3.3. Soluble Solid Content Prediction Performance Across Five Grape Cultivars

Building upon the superior classification performance demonstrated in the previous section, the regression task of soluble solid content (SSC) prediction was conducted to further evaluate the generalization capability and cross-modal learning effectiveness of the proposed DualStream-RTNet architecture. Using PCA-compressed spectral inputs, SSC prediction experiments were performed on five grape cultivars—Shine Muscat, Yubo No. 2, Longyan, Red Globe, and Meiren Zhi. To reduce the dimensionality and redundancy inherent in hyperspectral data, principal component analysis (PCA) was applied to the preprocessed spectral features prior to model input. Given the strong inter-band correlations typically observed in hyperspectral measurements, PCA provides an effective data-driven approach for compressing spectral information while preserving the dominant variance structure. In this study, the first six principal components were retained as input features, capturing the major spectral variability of the dataset while substantially reducing computational complexity. The performance of the proposed method was compared with several representative regression models, including PLSR, SVR, LSTM, and a Transformer-based model. The calibration (*R^2^c*, *RMSEc*) and prediction (*R^2^p*, *RMSEp*) results are summarized in Table 12.

Across all cultivars, traditional machine learning models such as PLSR and SVR exhibited moderate predictive ability, with PLSR achieving *R^2^p* values ranging from 0.7455 to 0.8369, and SVR showing slightly improved but still limited prediction performance. Although these models captured basic linear or nonlinear relationships within the spectral data, their relatively high *RMSEp* values indicate limited capability in modeling the complex spectral–chemical interactions underlying SSC variation.

Deep learning models demonstrated markedly enhanced performance. LSTM yielded improved prediction accuracy for most cultivars, with *R^2^p* values between 0.8461 and 0.8864, reflecting its ability to extract sequential dependencies within spectral signatures. The Transformer model further enhanced prediction stability, benefiting from its global attention mechanism, and achieved *R^2^p* values up to 0.9131 for Longyan and substantially reduced *RMSEp* values.

**Figure 9:**
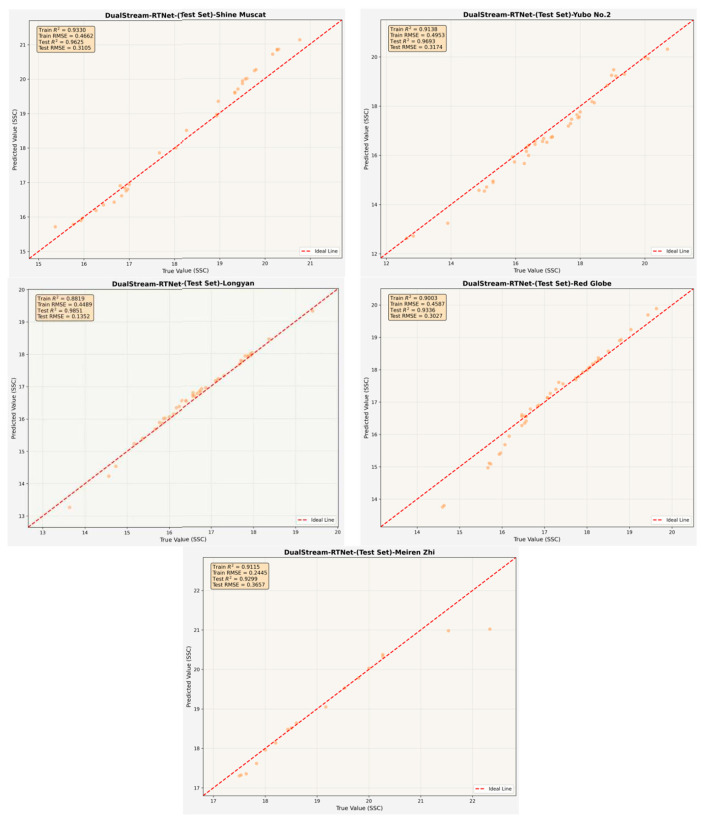
Regression Analysis of SSC Prediction Results Obtained by DualStream-RTNet.

Notably, DualStream-RTNet consistently outperformed all other models across nearly all grape cultivars, demonstrating its strong capacity for nonlinear spectral feature extraction and regression modeling. For instance, DualStream-RTNet achieved an *R^2^p* of 0.9693 for Yubo No. 2 (*RMSEp* = 0.3174), 0.9851 for Longyan (*RMSEp* = 0.1352), and 0.9336 for Red Globe (*RMSEp* = 0.3027). Even for cultivars with inherently higher SSC variability, such as Meiren Zhi, the model still delivered robust predictions (*R^2^p* = 0.9299). These results demonstrate that the dual-stream design and Transformer-enhanced feature fusion effectively capture both global and local spectral patterns, leading to more stable and accurate SSC estimation. The regression fitting curves of the predicted versus measured SSC values for the five grape cultivars using the DualStream-RTNet model are presented in Figure 9.

Overall, the SSC prediction experiments further validate the dual-task strength of the DualStream-RTNet framework. While the classification task verified the model’s discriminative ability using RGB-HSV images, the SSC prediction task demonstrates its strong regression capability using spectral inputs. Together, these findings confirm that DualStream-RTNet serves as a powerful and versatile architecture for multimodal fruit quality assessment, offering significant advantages over conventional machine learning and standard deep learning models.

### 3.4. Structural Contribution Analysis of Fusion and Multi-Task Learning

From the perspective of fusion strategy, the experimental results demonstrate a clear and progressive performance enhancement across different architectural configurations. When RGB images were used independently, the ResNet18 baseline achieved a test accuracy of 80.35%. Replacing RGB with HSV inputs led to a modest improvement to 82.08%, indicating that alternative color-space representations contribute complementary chromatic cues. Integrating RGB and HSV within a single-stream CNN framework further increased accuracy to 84.39%, confirming that multimodal color integration strengthens discriminative representation. The most substantial improvement was achieved after introducing the DualStream-RTNet architecture with learnable softmax-weighted fusion, where classification accuracy reached 93.64%. This stepwise performance evolution suggests that the observed gains are not attributable merely to feature concatenation, but rather to adaptive cross-stream interaction and enhanced global contextual modeling. The pronounced improvement from single-stream fusion to the dual-stream architecture highlights the structural effectiveness of the proposed fusion mechanism in capturing complementary chromatic–textural information.

A comparable structural advantage is evident in the SSC regression task. Relative to traditional regression approaches such as PLSR and SVR, as well as standalone deep learning models including LSTM and Transformer, the proposed framework consistently achieved higher R^2^p values and lower RMSEp across all five grape cultivars. This consistent superiority indicates that the dual-stream spectral modeling strategy effectively captures both localized spectral variations and long-range wavelength dependencies. The robustness of performance across cultivars with varying SSC distributions further suggests that the fusion architecture enhances nonlinear representation capacity rather than relying on increased parameterization.

With respect to multi-task learning, the unified framework demonstrates stable joint optimization of cultivar classification and SSC prediction without observable performance compromise in either task. Both classification accuracy and regression precision exceed those of their respective standalone baselines, indicating that shared representation learning contributes beneficial regularization and facilitates cross-task feature reinforcement. From a physiological perspective, grape color development and soluble solid accumulation are inherently coupled during the ripening process, providing a plausible biological basis for shared latent representations. Consequently, the multi-task architecture reduces structural redundancy while effectively leveraging intrinsic correlations between external phenotype and internal physicochemical attributes.

## 4. Discussion

The results obtained in this study collectively demonstrate that the proposed DualStream-RTNet architecture provides substantial advantages for both grape cultivar classification and soluble solid content (SSC) prediction. These dual-task improvements highlight the model’s ability to effectively integrate multimodal information and capture complementary feature representations, offering significant progress beyond conventional machine learning and single-stream deep learning models.

First, the superior classification performance observed across multiple experiments confirms the effectiveness of the dual-stream design for RGB-HSV image processing. By separately extracting discriminative features from two color spaces and subsequently integrating them through a Transformer-enhanced fusion mechanism, DualStream-RTNet successfully captures both local chromatic variations and global textural patterns. Traditional CNNs such as ResNet18, VGG16, and MobileNetV3 exhibit limited capacity in modeling subtle color differences among visually similar cultivars, and deeper architectures such as ResNet101 suffer from overfitting. In contrast, DualStream-RTNet achieves substantial gains in accuracy, precision, and recall, demonstrating that cross-space feature complementarity and global context modeling are crucial for fine-grained cultivar discrimination.

Second, the results of SSC prediction further underscore the versatility and robustness of the proposed architecture. Unlike the image-based classification task, SSC estimation relies on the nonlinear chemical–spectral relationship embedded within PCA-compressed hyperspectral data. Traditional regression models such as PLSR and SVR struggle to approximate these relationships, yielding limited predictive accuracy and relatively high RMSE values. Deep learning approaches such as LSTM and Transformer offer improved performance, but remain inconsistent across cultivars, indicating challenges in capturing complex spectral dependencies. DualStream-RTNet, however, achieves the most accurate and stable predictions across all five grape cultivars, demonstrating its strong capacity for nonlinear regression and spectral feature representation. These findings highlight that the dual-stream framework, combined with Transformer-based global attention, is highly effective not only for categorical discrimination but also for continuous trait prediction.

Importantly, the concurrent superiority of DualStream-RTNet in both tasks reflects its intrinsic multimodal adaptability. The architecture’s capacity to generalize across image and spectral domains suggests that it is not task-specific, but rather constitutes a unified framework suitable for a wide range of phenotyping applications. This characteristic aligns well with emerging trends in digital agriculture, where comprehensive quality assessment increasingly requires the integration of visual, spectral, and physicochemical information.

Furthermore, the present study advances existing research by demonstrating that a single deep learning framework can simultaneously excel in classification and regression tasks, which has been insufficiently explored in the context of fruit quality evaluation. Most previous studies have focused on either cultivar identification or quality parameter prediction in isolation, neglecting the potential synergy between multimodal information sources. By bridging this gap, DualStream-RTNet establishes a new perspective for designing unified, cross-modal phenotyping models and provides a promising direction for future research on intelligent fruit grading, automated quality monitoring, and high-throughput phenotyping systems.

Although DualStream-RTNet demonstrates strong performance in both classification and SSC prediction, several aspects offer room for further refinement. First, although the dataset was carefully collected and strictly controlled, it remains geographically constrained to a single grape-growing region and includes a limited number of cultivars. As grape phenotypic characteristics and internal quality attributes are influenced by environmental factors such as climate, soil conditions, and cultivation practices, the generalizability of the proposed model to other regions, seasons, or varieties may be limited. Second, all image and spectral data were acquired under laboratory-controlled conditions, ensuring high data consistency but not fully reflecting the variability encountered in real-world vineyard environments, such as complex backgrounds, illumination changes, and occlusions. Therefore, the present study primarily demonstrates the methodological feasibility and performance potential of the proposed multimodal multi-task framework rather than its immediate field deployability. Future work will focus on expanding the dataset across multiple growing regions and seasons, as well as validating the model under field-based and real-time acquisition scenarios, to further enhance robustness and practical applicability.

Overall, the findings affirm that DualStream-RTNet represents a significant methodological advancement with clear practical implications. Its strong generalization ability, multimodal adaptability, and dual-task performance potential make it a compelling candidate for broader application in precision agriculture and postharvest quality assessment.

## Figures and Tables

**Table 1:** Sample Allocation of Five Grape Cultivars in the Training and Test Sets.

Name	Training Set	Test Set	Total
C1 (Shine Muscat)	131	33	164
C2 (Yubo No. 2)	160	40	200
C3 (Longyan)	163	41	204
C4 (Red Globe)	170	42	212
C5 (Meiren Zhi)	67	17	84
Total	691	173	864

**Table 2:** Extracted RGB Statistical Features of Grape Berry Images.

Name	R_MEAN	G_MEAN	B_MEAN	R_STD	G_STD	B_STD
C1-1	0.521604	0.52094495	0.493599	0.08721417	0.09194911	0.1643389
C1-2	0.50284606	0.500796	0.48493525	0.07070318	0.076193325	0.12683493
…	……					
C5-82	0.4909724	0.47594947	0.4769472	0.08039917	0.123555206	0.13555987
C5-83	0.5177455	0.50448185	0.50452876	0.10641602	0.14601436	0.14255233
C5-84	0.49686614	0.48555347	0.4844106	0.07312628	0.11181258	0.1235537

**Table 3:** Average values RGB Statistical Features of Five Grape Cultivars.

Name	R_MEAN	G_MEAN	B_MEAN	R_STD	G_STD	B_STD
C1-Avg	0.51050387	0.51151363	0.48875176	0.08837727	0.08563126	0.15013374
C2-Avg	0.50670495	0.50241094	0.48013962	0.08126136	0.09027932	0.15229831
C3-Avg	0.49671971	0.49172323	0.48796102	0.08480384	0.10488056	0.11319816
C4-Avg	0.51132961	0.50641911	0.50732869	0.10663285	0.12973809	0.12856674
C5-Avg	0.51079533	0.48578837	0.48322816	0.09961931	0.15578151	0.16100695

**Table 4:** Extracted HSV Statistical Features of Grape Berry Images.

Name	H_MEAN	S_MEAN	V_MEAN	H_STD	S_STD	V_STD
C1-1	0.27691898	0.10592300	0.52592796	0.27465886	0.26313720	0.08797890
C1-2	0.12339456	0.06465100	0.50410880	0.22839811	0.21486822	0.07055739
…	……					
C5-82	0.47185448	0.08831242	0.50029200	0.25757122	0.18703671	0.08308102
C5-83	0.26926480	0.06896078	0.52041143	0.32814565	0.18891947	0.10716134
C5-84	0.38073212	0.07295054	0.50449310	0.27031276	0.17494366	0.07447323

**Table 5:** Average values for the HSV Statistical Features of Five Grape Cultivars.

Name	H_MEAN	S_MEAN	V_MEAN	H_STD	S_STD	V_STD
C1-Avg	0.26672694	0.09670954	0.51650546	0.25959151	0.24580692	0.08589040
C2-Avg	0.33960341	0.10729508	0.51295989	0.27414749	0.23640034	0.08299419
C3-Avg	0.30245830	0.05389950	0.50168218	0.26953734	0.12890331	0.08586215
C4-Avg	0.27098909	0.05448948	0.51664020	0.27005138	0.14209165	0.10787342
C5-Avg	0.35615054	0.10902063	0.51587458	0.30707264	0.22289020	0.10139210

**Table 6:** SSC Measurements of Grape Samples Across Cultivars.

Name	SSC
C1-1	20.56666667
C1-2	20.53333333
…	…
C5-82	22.33333333
C5-83	19.76666667
C5-84	19.5

**Table 7:** Performance Comparison of Different CNN Models for Grape Cultivar Classification Using RGB Inputs.

Models	Training Set			Test Set		
	Accuracy (%)	Precision (%)	Recall (%)	Accuracy (%)	Precision (%)	Recall (%)
ResNet18	81.48	83.20	79.88	80.35	84.05	80.35
MobileNetv3	84.52	81.06	74.38	69.94	75.16	69.94
Vgg16	68.89	68.84	68.89	76.88	78.93	76.88

**Table 8:** Performance Comparison of Different CNN Models for Grape Cultivar Classification Using HSV Inputs.

Models	Training Set			Test Set		
	Accuracy (%)	Precision (%)	Recall (%)	Accuracy (%)	Precision (%)	Recall (%)
ResNet18	76.27	86.13	85.67	82.08	82.36	82.08
MobileNetv3	78.87	82.72	79.16	76.30	80.77	76.30
Vgg16	64.40	64.31	64.40	75.14	79.34	75.14

**Table 9:** Performance Comparison of CNN Models for Grape Cultivar Classification Using Fused RGB-HSV Inputs.

Models	Training Set			Test Set		
	Accuracy (%)	Precision (%)	Recall (%)	Accuracy (%)	Precision (%)	Recall (%)
ResNet18	85.09	92.43	92.19	84.39	84.96	84.39
MobileNetv3	91.46	89.47	83.94	80.92	86.69	80.92
Vgg16	82.05	82.11	82.05	80.35	84.64	80.35

**Table 10:** Effect of Network Depth on Grape Cultivar Classification Performance Using RGB-HSV Fused Inputs.

Models	Training Set			Test Set		
	Accuracy (%)	Precision (%)	Recall (%)	Accuracy (%)	Precision (%)	Recall (%)
ResNet18	85.09	92.43	92.19	84.39	84.96	84.39
ResNet34	80.32	88.33	87.26	83.24	85.37	83.24
ResNet50	79.88	87.54	86.11	85.55	87.09	85.55
ResNet101	85.67	82.02	76.56	73.41	80.93	73.41

**Table 11:** Performance Comparison Between DualStream-RTNet and ResNet18 Using RGB-HSV Fused Inputs.

Models	Training Set			Test Set		
	Accuracy (%)	Precision (%)	Recall (%)	Accuracy (%)	Precision (%)	Recall (%)
ResNet18	85.09	92.43	92.19	84.39	84.96	84.39
DualStream-RTNet	92.91	96.12	95.95	93.64	93.90	93.64

**Table 12:** SSC Prediction Performance of DualStream-RTNet Compared with Conventional and Deep Learning Regression Models.

	Variety	Shine Muscat				Yubo No. 2				Longyan				Red Globe				Meiren Zhi			
Models		*R^2^_c_*	*RMSE_c_*	*R^2^_p_*	*RMSE_p_*	*R^2^_c_*	*RMSE_c_*	*R^2^_p_*	*RMSE_p_*	*R^2^_c_*	*RMSE_c_*	*R^2^_p_*	*RMSE_p_*	*R^2^_c_*	*RMSE_c_*	*R^2^_p_*	*RMSE_p_*	*R^2^_c_*	*RMSE_c_*	*R^2^_p_*	*RMSE_p_*
PLSR		0.802	0.809	0.7455	0.7753	0.9293	0.4605	0.7961	0.7241	0.9408	0.3267	0.8369	0.3412	0.8498	0.5315	0.7825	0.6983	0.8829	0.3331	0.7605	0.4588
SVR		0.862	0.675	0.769	0.746	0.837	0.7	0.795	0.739	0.782	0.596	0.769	0.594	0.76	0.703	0.764	0.606	0.84	0.394	0.771	0.404
LSTM		0.9298	0.4772	0.8718	0.5743	0.9232	0.4675	0.8864	0.6107	0.8229	0.5498	0.8573	0.4183	0.9049	0.448	0.8461	0.461	0.8024	0.3653	0.8495	0.5358
Transformer		0.889	0.57	0.92	0.537	0.928	0.468	0.893	0.503	0.934	0.346	0.913	0.249	0.833	0.585	0.884	0.438	0.795	0.452	0.854	0.31
DualStream-RTNet		0.933	0.4662	0.9625	0.3105	0.9138	0.4953	0.9693	0.3174	0.8819	0.4489	0.9851	0.1352	0.9003	0.4587	0.9336	0.3027	0.9115	0.2445	0.9299	0.3657

## Data Availability

The raw data supporting the conclusions of this article will be made available by the authors on request.
